# Genetic diversity of *Phytophthora infestans *in the Northern Andean region

**DOI:** 10.1186/1471-2156-12-23

**Published:** 2011-02-09

**Authors:** Martha Cárdenas, Alejandro Grajales, Roberto Sierra, Alejandro Rojas, Adriana González-Almario, Angela Vargas, Mauricio Marín, Gustavo Fermín, Luz E Lagos, Niklaus J Grünwald, Adriana Bernal, Camilo Salazar, Silvia Restrepo

**Affiliations:** 1 Universidad de Los Andes, Bogotá D.C., Colombia; 2 Universidad Nacional, Medellín, Antioquia, Colombia; 3 Universidad de Los Andes, La Hechicera, Mérida, Venezuela; 4 Universidad de Nariño, Pasto, Nariño, Colombia; 5 USDA Agricultural Research Service, Corvallis, OR, USA; 6 Smithsonian Tropical Research Institute. Apartado 0843-03092, Panamá, República de Panamá; 7 Department of Zoology, University of Cambridge, Downing Street, Cambridge, CB2 3EJ, UK

## Abstract

**Background:**

*Phytophthora infestans *(Mont.) de Bary, the causal agent of potato late blight, is responsible for tremendous crop losses worldwide. Countries in the northern part of the Andes dedicate a large proportion of the highlands to the production of potato, and more recently, solanaceous fruits such as cape gooseberry (*Physalis peruviana*) and tree tomato (*Solanum betaceum*), all of which are hosts of this oomycete. In the Andean region, *P. infestans *populations have been well characterized in Ecuador and Peru, but are poorly understood in Colombia and Venezuela. To understand the *P. infestans *population structure in the Northern part of the Andes, four nuclear regions (ITS, *Ras*, *β-tubulin *and *Avr3a*) and one mitochondrial (*Cox1*) region were analyzed in isolates of *P. infestans *sampled from different hosts in Colombia and Venezuela.

**Results:**

Low genetic diversity was found within this sample of *P. infestans *isolates from crops within several regions of Colombia and Venezuela, revealing the presence of clonal populations of the pathogen in this region. We detected low frequency heterozygotes, and their distribution patterns might be a consequence of a high migration rate among populations with poor effective gene flow. Consistent genetic differentiation exists among isolates from different regions.

**Conclusions:**

The results here suggest that in the Northern Andean region *P. infestans *is a clonal population with some within-clone variation. *P. infestans *populations in Venezuela reflect historic isolation that is being reinforced by a recent self-sufficiency of potato seeds. In summary, the *P. infestans *population is mainly shaped by migration and probably by the appearance of variants of key effectors such as *Avr3a*.

## Background

The potato and tomato late blight pathogen, *Phytophthora infestans *(Mont.) de Bary [1], has a broad host range within the Solanaceae family including *Solanum phureja *(yellow potato), *S. betaceum *(tree tomato), *S. quitoense *(naranjilla or lulo), *Physalis peruviana *(cape gooseberry), and other wild species [2,3]. Since the Irish potato famine in the 19th century, this pathogen has been thoroughly studied because of its severe economic impact on agriculture, causing billion-dollar losses annually [4].

Mitochondrial and nuclear DNA regions of *P. infestans *have been extensively used in different regions of the world to investigate its evolutionary history and population structure [5-7]. This has led to the definition of lineages on the basis of molecular data, which has allowed the monitoring of populations and resulted in the generation of important epidemiological inferences about the pathogen's migration [5,6]. Additionally, population studies of *P. infestans *have been conducted to elucidate the origin and diversity of isolates from cultivated species [5,8-10] and to determine if there are any differences between populations from wild and cultivated hosts [11-13].

Population studies of *P. infestans *in the Andean region have been focused on Peru and Ecuador. These two countries are considered to be the center of origin of potatoes, [14] and thus some authors have proposed that this region is also the center of origin of the *P. infestans *pathogen [8,9]. In Ecuador, the pathogen has shown low levels of diversity, and its population structure is strongly influenced by host preference. Each *P. infestans *clonal lineage is associated with a different host group: US-1 lineage with tomato, EC-1 with potato, EC-2 with wild solanaceous species, particularly with the *Anarrhichomenum *section, and EC-3 with *S. betaceum *[15-17]. Additionally, genetic differentiation is found among isolates of *P. infestans *associated with *S. ochranthum *[18]. Geographically, no clear subdivision was found in Ecuador when using allozymes and RFLP markers [15]. A different pattern was obtained in Peru, where the lineages are neither structured according to any host species [19] nor according to the origin of the hosts, be they cultivated or wild [20]. Other population studies have documented higher diversity in Peru than in Ecuador, according to the number of genotypes found [11]. According to mitochondrial haplotypes, *P. infestans *has been classified into four main groups: Ia, Ib, IIa and IIb [7,21,22]. Each mitochondrial haplotype has been used to complement the definition of *P. infestans *lineages. The US-1 lineage has been related to the Ib haplotype, the US-6 to the IIb haplotype, the EC-1 to the IIa haplotype and the EC-2 and EC-3 lineages to the Ia haplotype. Recently a new mitochondrial haplotype, Ic, has been also related to lineage EC-2, and has been correlated with a new species, *P. andina *[8,23].

Colombia is considered to be the fourth largest potato producer in Latin America with a production of 1.9 million tons and a cultivated surface of 110,000 hectares in 2007 [24]. However, *P. infestans *population studies are scarce [25,26]. These studies showed that the *P. infestans *population revealed low genetic diversity, with no evidence of sexual reproduction, and consisted mostly of the A1 mating type. It should be noted that the A2 mating type was recently detected, but only in one isolate [26], and a posterior intensive sampling in this site could not detect the A2 mating type again [26]. To this date, only one study has investigated the population structure of *P. infestans *in Venezuela [27]. Amongst the sample over this time period, only A1 mating type was reported.

This is the first study that makes use of nuclear and mitochondrial regions to conduct a population genetic analysis of the pathogen in the North Andean region. Furthermore, sequence diversity at the *Avr3a *avirulence (effector) gene was examined to investigate whether populations are subject to selection at this locus. Only two alleles have been described for the *Avr3a *locus according to virulence and avirulence against the *S. demissum *R3a gene [28]. Since effector genes affect pathogenicity and fitness, they may be under specific selective pressures after mutation dissemination through genetic drift, responding to host populations or other local factors. Local adaptation seems to be playing an important role in the diversification and distribution of the effectors alleles/haplotypes in natural and agricultural environments [29]. Thus this locus could be informative about the *P. infestans *adaptive history.

Our goals were to conduct sequencing of different genic regions for populations of *P. infestans *from the Northern Andes to describe the geographical distribution of diversity. We examined genes likely to be selectively neutral or under selection pressure in order to evaluate the importance of selection, drift and gene flow in specific genic regions. Additionally, these analyses are useful in comparing population parameters of the Northern Andes populations of the pathogen with other populations previously described in different regions worldwide.

## Methods

### Strains and Culture Conditions

A total of 80 strains from Colombia and Venezuela were included in this study (Additional files 1 and 2). Sixty-eight Colombian strains were obtained from different states, including Cundinamarca (N = 23), Antioquia (N = 18), Nariño (N = 15) and Boyacá (N = 12), isolated from *S. tuberosum*, *S. phureja*, *S. lycopersicum*, *S. betaceum*, *S. quitoense*, and *P. peruviana*. All the strains from Cundinamarca and seven from Antioquia were previously characterized using mitochondrial haplotype, metalaxyl resistance, isoenzymes analysis (Gpi and Pep) and restriction fragment length polymorphism with probe RG57 (Additional file 1) [26]. Venezuelan strains (N = 12) were selected from a collection of more than 500 strains isolated from *S. tuberosum *[27] from the three Andean states of Mérida, Táchira and Trujillo. All isolates were collected from commercial crops. All the isolates were identified as *P. infestans sensu lato *by ITS sequencing. Additionally, eight isolates from Ecuador, kindly provided by Michael Coffey, were also included for the diversity analysis. Isolates US940480 (mating type A2) and US940494 (mating type A1), provided by William E. Fry (Cornell University), were used as reference strains. In general, all isolates were cultured on rye medium and incubated at 18°C for eight days in the dark [30].

### DNA Amplification and Sequencing

DNA extraction was performed as previously described [31]. Four nuclear loci, *ITS*, *β-tubulin*, *Ra*s and *Avr3a*, as well as one mitochondrial gene, cytochrome c oxidase 1 (*Cox1*) were amplified and sequenced. For ITS, Internal Transcribed Spacer 1, 5.8 S rDNA and Internal Transcribed Spacer 2 were amplified using ITS4 and ITS5 primers yielding a 950 bp product [32]. Primers TUBUF2 and TUBUR1 were used to amplify a 989-bp portion of the *β-tubulin *gene, excluding introns [33]. The *Ras *sequence was obtained after two independent amplifications. IRF and IRR primers were used to amplify a 223-bp region corresponding to intron 1. Additionally, RASF and RASR primers amplified a 600-bp product corresponding to partial exons 3 and 6, complete exons 4 and 5, and complete introns 3 to 5 [8,34]. *Avr3a *was obtained after modification of previously described primers [28] with an M13 tail to allow for amplification and sequencing of 453 bp corresponding to the entire gene (M13Pex F: 5'-GTAAAACGACGGCCAGCCATGCGTCTGGCAATTATGCT-3' and M13Pex R: 5'-CAGGAAACAGCTATGACCTGAAAACTAATATCCAGTGA-3'). COXF4N and COXR4N primers were employed to amplify 972 bp of the mitochondrial gene *Cox1 *[33].

For all primer combinations the reaction conditions were the following, in a final volume of 25 μl: 1× PCR buffer, 0.5 mM dNTPs, 2.5 mM MgCl2, 0.2 mM of each primer, 1U Taq DNA polymerase. PCR conditions for ITS and *Ras *started with an initial denaturation step of 96°C for 2 min (1 min for *Ras*), 35 cycles of 96°C for 1 min, 55°C for 1 min (56°C for *Ras*), 72°C for 2 min, and a final step of 10 min at 72°C. For *β-tubulin *and *Cox*1, the conditions started with a denaturation step of 2 min at 94°C, followed by 35 cycles of 94°C for 30 s (1 min for *Cox*1), 60°C for 30 s (45 s for *Cox1*) 72°C for 1 min and a final step of 10 min at 72°C. For *Avr3a*, the amplification conditions included a denaturation step of 2 min at 94° C followed by 15 cycles of 30 s at 94°C, 30 s at 55°C and 1 min at 72°C. Twenty-five additional cycles of 30 s at 94°C, 30 s at 62°C and 1 min at 72° C were added. The final extension was performed for 10 min at 72°C. The amplification products were separated on 1% agarose gels and visualized by staining with ethidium bromide. Single band PCR products were sequenced in Macrogen, Korea. Sequence assembly and editing was performed manually on the CLC DNA Workbench http://www.clcbio.com. Sequence alignments were performed using MUSCLE [35]. Due to the high heterozygosity condition of *P. infestans*, double peaks were assigned with the IUPAC code when necessary.

Haplotype reconstruction was conducted using DNAsp v4.90.1 [36] implementing the algorithm provided in PHASE [37]. Basically, PHASE assigns a probability of the correct inference of haplotype phase at every heterozygous position. PHASE simulations were repeated four times for each locus, two without recombination and two with recombination. Each simulation was run with 5,000 iterations. In all cases, the most common output inferring haplotypes with >95% confidence was accepted. All the haplotypes sequences were deposited in GeneBank under accession numbers [GenBank: GU258154 and GU258156] for *Ras*, [GenBank:GU258157-GU258165; GU258167, GU258168] for *β-tubulin*, [GenBank: GU258058 and GU258059] for *Cox1 *and [GenBank:GU258052-GU258057] for Avr3a. Additionally, sequences for ITS were deposited for all the strains employed under accession numbers [GenBank:GU258061-GU258072, GU258074-GU258077, GU258080-GU2580125, GU2580127-GU2580152].

### Gene Networks and Population Genetic Analyses

Different datasets were generated for each DNA region. Additional sequences of ITS, *β-tubulin*, *Ras *and *Cox*1, from *P. infestans *isolates collected worldwide were retrieved from GenBank and included in the analyses (additional file 3). The genealogical relationships among haplotypes were established by statistical parsimony with a 95% connection limit as is implemented in the TCS software version 1.21 [38].

Nucleotide diversity (π), nucleotide substitution rate (θ) and haplotypic diversity (HD) were calculated for each data set from the Northern Andean region, including Colombia and Venezuela (NA) and for the rest of the world, corresponding to available sequences from other geographical origin (R) using DnaSP v.4.90.1 [36]. For the worldwide data set (W) the combination of the NA and R data sets was used (W = NA +R. The population genetics analyses were performed only with the NA dataset.

To test the hypothesis of genetic structure (complete Panmixia vs. geographically structured populations) among these populations, a permutation test for Hudson's statistics [39] was performed with SNAP Workbench [40]. Sequences were collapsed into haplotypes recoding indels and excluding infinite-sites violations using MAP TOOL [40]. Then, a distance matrix was generated with SEQTOMATRIX [40]. This matrix was used to perform a non-parametric permutation test with PERMTEST [40] under default parameters.

Tajima's D [41] was used as a test of neutral evolution for β*-tubulin*, *Ras, Avr3a *and *Cox*1 with DnaSP v.4.90.1 [36]. A mismatch distribution analysis was carried out with the statistic R2 [42] to establish population size changes as is implemented in DnaSP v.4.90.1 [36]. To assess a confidence interval for R2 value observed, a coalescent simulation with 1000 replicates was run with theta (θ) and no recombination as starting parameters.

The software SITES [43] was used to estimate the number of fixed differences or shared polymorphisms among the defined populations in the NA dataset, using *β-tubulin*, *Ras *and *Cox*1. Fixed differences would be indicative of isolation, while shared polymorphisms would indicate gene flow. MIGRATE-n [44] was used to estimate theta and the direction and amount of gene flow among these populations, with nuclear *(β-tubulin*, *Ras*) and mitochondrial (*Cox*1) genes. In each case, 20 short chains with 5000 sampled genealogies and five short chains with 50000 genealogies were run. Heating was set to be active with four temperatures (1.0, 1.5, 2.5 and 3.0).

A pairwise comparison of the rates of nonsynonymous nucleotide substitutions per nonsynonymous site (dN) and synonymous nucleotide substitutions per synonymous site (dS) were estimated for *Avr3a *using the approximate method of Nei and Gojobori (1986) [45] implemented in the YN00 program in PAML [46]. CODEML was employed to identify the amino acids under strong diversifying selection. *Avr3a *sequences were translated into amino acids and aligned using *P. sojae *[GenBank: EF463064] as an outgroup. Details of these tests are explained elsewhere [47].

## Results

### Summary statistics and Gene Networks

**Figure 1:**
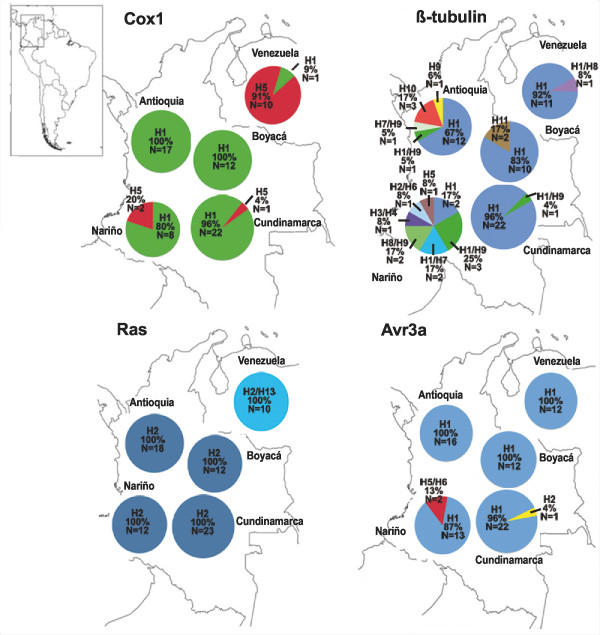
***Cox1*, *β-tubulin*, *Ras *and *Avr3a *haplotype distribution of *Phytophthora infestans *in the Northern Andean region**. Colors indicate the different haplotypes for each locus analyzed.

Tables 1 and 2 contain the summary statistics for the analyzed sequences and haplotype information. The number of sequences analyzed differed for each gene, due to challenges with the amplification/sequencing of specific isolates or availability of previously published data (Table 1). The ITS region showed no polymorphism in any of the 755 sites analyzed and was excluded from further analyses (and from Table 1). The Northern Andean region (Venezuela and Colombia) showed low levels of nucleotide diversity and genetic variability (Table 1). The regional distribution of the haplotypes is shown in Figure 1. The highest number of haplotypes was observed for *β-tubulin*. The haplotype most frequently found (H1) was present in Colombia and Venezuela. Venezuelan *P. infestans *showed only two haplotypes, H1 and H8. The Central Andes showed four haplotypes, one of them restricted to this zone (H10). In the Eastern Andes three haplotypes were found for *β-tubulin*, and H11 was only found in this region. The Southwestern Andes showed the highest number of different haplotypes for this gene (9), five of them (H2, H3, H4, H5 and H6) restricted to this area. For *Ras*, haplotype the same nomenclature was used as the one in Gomez-Alpizar *et al*., 2007 [8] for comparison purposes. The *Ras *region showed two haplotypes (H2 and H13) with only one haplotype (H2) found in the Southwestern, Central and Eastern Andes. Venezuelan isolates were heterozygotic (H2/H13) and the H13 haplotype was exclusive to this country. *Cox1 *showed two haplotypes (H1 and H5), and H1 was present in all regions. All isolates from the Central Andes belonged to only this haplotype, which was also the most common one in the Southwestern and Eastern Andes. H5 was the most common haplotype in Venezuela (Figure 1). For *Avr3a*, four haplotypes were found. The most frequent allele, H1, was present in Colombia and Venezuela. H5 and H6 were only found in the Southwestern Andes. H2 was only found in the Eastern Andes. All isolates from *S. tuberosum *corresponded to haplotype H1, which was the most common haplotype. In *S. betaceum *the main haplotypes were H1, H2, H5 and H6 (Additional file 1).

**Table 1:** Summary statistics for loci *Cox1*, *Ras*, ITS, *β-tubulin *and *Avr3a *from *P. infestans *in the Northern Andean region and worldwide.

GEOGRAPHIC REGION	LOCUS	Sequences (N) ^a^	Length (bp)	Segregating SITES	GENETIC VARIABILITY (Segregating Sites/Length)	π^b^	θ SITE ^c^	N Haplotypes	HD^d^
NORTHERN ANDEAN (Colombia and Venezuela)	*Cox1*	73	669	1	0.15	0.0004	0.0003	2	0.3
	*Ras*	85	765	3	0.39	0.0008	0.0008	2	0.21
	*β-tub*	90	815	14	1.72	0.0015	0.0036	11	0.47
	*Avr3a*	80	444	12	2.70	0.0017	0.0055	4	0.12

Central Andes (Antioquia)	*Cox1*	17	669	0	0	0	0	1	0
	*Ras*	18	765	0	0	0	0	1	0
	*β-tub*	20	815	4	0.49	0.0012	0.0014	4	0.56
	*Avr3a*	16	444	0	0	0	0	1	0

Eastern Andes (Boyacá Cundinamarca)	*Cox1*	35	669	1	0	0.00009	0.0004	2	0.057
	*Ras*	35	765	0	0	0	0	1	0
	*β-tub*	36	815	2	0.24	0.0002	0.0006	3	0.16
	*Avr3a*	35	444	1	0.22	0.0001	0.0005	2	0.057

Southwestern Andes (Nariño)	*Cox1*	10	669	1	0.15	0.0005	0.0005	2	0.36
	*Ras*	12	765	0	0	0	0	1	0
	*β-tub*	21	815	12	1.47	0.004	0.004	9	0.843
	*Avr3a*	17	444	11	2.48	0.007	0.007	3	0.412

Venezuela	*Cox1*	11	669	1	0.15	0.0003	0.0005	2	0.182
	*Ras*	20	765	3	0.39	0.0021	0.0011	2	0.526
	*β-tub*	13	815	1	0.13	0.0002	0.0004	2	0.154
	*Avr3a*	12	444	0	0	0	0	1	0

REST OF THE WORLD	*Cox1*	69	669	4	0.59	0.0097	0.0012	5	0.574
	*Ras*	166	766	13	1.69	0.0031	0.0029	12	0.583
	*β-tub*	13	815	2	0.24	0.0011	0.0008	3	0.59
	*Avr3a*	8	444	4	0.9	0.0046	0.0035	3	0.607
	*Cox1*	142	669	4	0.59	0.00078	0.0011	5	0.473

**WORLDWIDE**	*Ras*	251	766	13	1.69	0.0024	0.0028	13	0.482
	*β-tub*	103	815	14	1.71	0.0016	0.0033	11	0.544
	*Avr3a*	88	444	12	2.7	0.002	0.0054	6	0.174

^a ^Numbers represent sequences and can exceed the numbers of strains due to heterozygous individuals

^b ^π = Nucleotide Diversity

^c ^θ SITE = Watterson's Theta per site

^d ^HD = Haplotype diversity

Colombia and Venezuela are designated NA; the rest of the world, corresponding to available sequences from other geographical origins, is designated R; and worldwide, corresponding to the combination of the two previous designations, is labeled W (W = NA +R).

**Table 2:** Haplotype distribution of *P. infestans *for *Avr3a*, *Ras*, *β-tubulin *and *Cox1*.

						Avr3a															Ras															β-tubulin											Cox 1			
Position					1	2	2	2	2	2	2	2	3	4					1	1	1	2	3	3	4	4	4	5	6						1	3	4	4	5	5	7	8	8	8				4	4	6
			4	5	8	0	0	2	3	4	6	8	0	1			6	9	0	1	6	0	1	4	6	7	9	8	3				3	6	3	5	2	7	2	4	7	0	0	1			1	2	4	2
			3	5	3	0	2	0	8	2	4	4	9	5			3	8	5	2	2	3	2	8	2	1	7	7	5			6	0	3	2	5	0	4	8	9	4	1	2	5			1	5	6	0
																																																		
Site												1	1	1												1	1	1	1												1	1	1	1						
Number			1	2	3	4	5	6	7	8	9	0	1	2			1	2	3	4	5	6	7	8	9	0	1	2	3			1	2	3	4	5	6	7	8	9	0	1	2	3			1	2	3	4
																																																		
Consensus			A	T	A	A	C	A	A	G	G	A	T	C			C	G	A	A	A	G	G	G	G	T	G	T	T			C	C	C	A	C	T	G	C	T	T	C	A	T			G	A	G	C
Site Type			v	v	v	v	t	t	t	t	v	t	v	v			t	v	v	t	t	t	v	t	v	t	t	t	v			t	t	t	t	t	t	t	t	v	t	t	v	v			v	t	v	t
Character Type			_	i	_	_	_	_	i	_	_	_	i	i			i	-	i	i	i	i	i	i	i	i	i	i	i			i	i	i	i	i	i	i	i	i	i	-	-	i			-	-	-	-
	H1	(80)	.	A	.	.	.	.	G	.	.	.	G	A	H1	(19)	T	T	C	G	.	A	.	.	.	.	A	C	A	H1	(68)	.	.	.	.	.	.	.	T	.	C	.	.	.	H2	(1)	C	.	.	.
	H2	(1)	T	A	.	.	.	.	G	.	.	.	G	A	H2	(178)	.	.	.	.	.	.	.	.	.	.	.	.	.	H2	(1)	T	T	T	G	T	.	.	.	G	.	.	.	.	H1	(94)	.	.	.	T
	H3	(2)	.	.	.	.	.	.	.	.	.	.	.	.	H3	(2)	.	.	.	.	.	.	T	A	.	C	.	.	.	H3	(1)	T	T	.	.	.	.	.	.	.	C	.	.	G	H5	(43)	.	.	.	.
	H4	(1)	.	.	.	.	.	.	.	.	.	.	.	A	H4	(14)	.	.	.	.	.	.	.	A	.	.	.	.	.	H4	(1)	T	T	T	G	T	C	A	.	G	.	.	.	G	H4	(1)	.	.	C	.
	H5	(2)	.	.	T	C	.	.	.	.	.	.	.	.	H5	(1)	.	.	.	.	.	.	.	A	T	C	.	.	.	H5	(1)	.	.	.	G	.	C	A	.	.	.	.	.	.	H3	(3)	.	G	.	.
	H6	(2)	.	.	.	.	T	G	.	A	T	G	.	.	H6	(20)	.	.	.	.	.	.	.	A	.	C	.	.	.	H6	(1)	.	.	.	.	T	.	.	.	.	.	.	.	.						
															H7	(1)	.	.	.	.	.	.	.	.	.	.	.	C	.	H7	(11)	.	.	.	.	.	.	.	.	.	.	.	.	.						
															H8	(1)	T	.	C	G	.	A	.	.	.	.	A	C	A	H8	(3)	.	.	.	.	.	.	.	.	.	C	.	.	.						
															H9	(1)	.	.	.	.	G	.	.	.	.	.	.	.	.	H9	(11)	.	.	.	.	.	.	.	T	.	.	.	.	.						
															H10	(1)	.	.	.	.	G	.	.	A	.	C	.	.	.	H10	(3)	.	.	.	.	.	.	.	T	.	C	T	C	.						
															H11	(1)	.	.	.	.	G	.	.	A	T	.	.	.	.	H11	(2)	.	.	.	.	.	.	.	T	.	C	.	.	G						
															H12	(2)	.	.	.	.	.	.	T	.	.	.	.	.	.																					
															H13	(10)	.	.	.	.	.	.	.	.	.	.	A	C	A																					

t: transitions; v: transversions; i: informative sites; -: uninformative sites; H: Haplotype.

Gene Networks were constructed for each Northern Andean region and for Colombia and Venezuela when more than one haplotype was found, in order to determine the evolutionary relationship among them (Additional files 4 and 5). As a general pattern, nuclear and mitochondrial haplotypes present in the Eastern and Central Andes as well as in Venezuela were only separated from each other by one or two steps. However, in the Southwestern Andes a different pattern was observed for *β-tubulin *and *Avr3a*. Three haplotypes were found for *Avr3a*: H1, H5 and H6. H5 and H6 were six and nine steps distant from H1, respectively. For *β-tubulin*, nine haplotypes were found and only five of them were related to each other by one step. More than two steps separated the other four haplotypes from each other.

**Figure 2:**
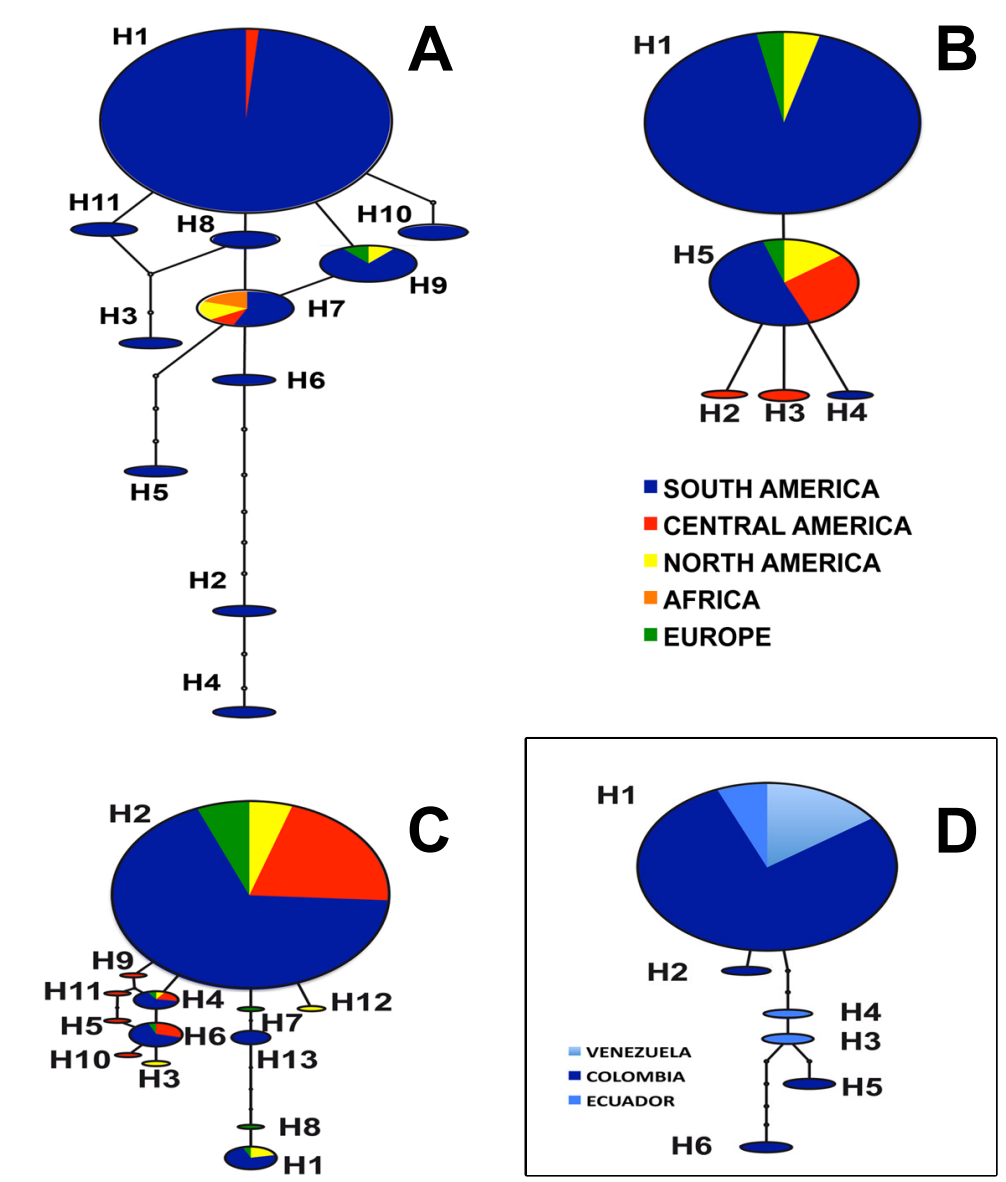
**Haplotype networks of genes studied in the *Phytophthora infestans *populations of the Northern Andean region**. **A**. *β-tubulin*; **B**. *Cox1*; **C**. *Ras*; **D**. *Avr3a*. Respective haplotypes are identified with the letter H, followed by a corresponding number. Sizes of the circles reflect haplotype frequencies in the population and were constructed with statistical parsimony as is implemented in TCS [38]. For *Ras*, South America: Colombia, Venezuela, Ecuador, Bolivia, Brazil, Peru; Central America: Mexico and Costa Rica; North America: USA; Europe: Ireland. For *β-tubulin*, South America: Colombia, Venezuela and Ecuador; Central America: Mexico; North America: Canada; Europe: The Netherlands; Africa: Uganda and Kenya. For *Cox1*, South America: Colombia, Venezuela, Ecuador, Bolivia, Brazil and Peru; Central America: Mexico and Costa Rica; North America: USA; Europe: Ireland.

Nucleotide diversity (π) and substitution rate were low in all genes analyzed in the R and W datasets (Table 1). Again, datasets were designated NA for Colombia and Venezuela, R for the rest of the world, which corresponds to available sequences from other geographical origins, and W for worldwide, corresponding to the combination of the two previous datasets (W = NA +R). The Northern Andean region showed the same pattern of low nucleotide diversity and genetic variability as the rest of the world population (Table 1). Haplotypic diversities were high as a consequence of heterozygous individuals showing alleles with low frequency. *β-tubulin *showed the highest number of haplotypes, followed by *Avr3a*, *Ras *and *Cox1 *(Table 1 and Figure 2). The Northern Andean region shared with the rest of the world three haplotypes for *β-tubulin *(H1, H7, H9), two haplotypes for *Cox1 *(H1 and H5), and just one haplotype for *Ras *(H2) and *Avr3a *(H1).

The estimated gene networks for the W dataset (Figure 2) showed as a general pattern a dominant haplotype separated by few steps from the others. The network representing the *β-tubulin *haplotypes showed a dominant haplotype, H1, present in Central America and South America (Figure 2A). H7 was present in Central America, South America, North America, and Africa. H9 was found in North and South America, as well as in Europe (The Netherlands). The remaining haplotypes were restricted to South America. The most divergent clade was represented by isolates from *S. betaceum *(data not shown). For *Cox*1, two haplotypes (H1 and H5) were widely distributed among regions and connected directly in the network. The three remaining haplotypes were derived from H5; two of them were restricted to Central America (Mexico) and one, H4, to South America (Ecuador) (Figure 2B). For the *Ras *gene, a dominant haplotype H2 was present in North America, Central America and South America, as well as in Europe (Ireland) (Figure 2C). H2 was mainly associated with *S*. *tuberosum*, although this species also hosted other haplotypes. H13 was only found in South America (Venezuela).

### Population Genetic Analyses

To test the hypothesis of population subdivision, isolates were first assigned to populations following their geographical distribution on a regional scale. Then, permutation tests for Hudson's statistics were performed for all combinations among regions (Table 3). Significant genetic differentiation was found for *Ras *and *Cox1*, between the Eastern Andes and Venezuela and between the Central Andes and Venezuela (Table 4). For *β-tubulin*, population subdivision was found between the Central and Eastern Andes and between the Southwestern and Eastern Andes (Table 3). *Avr3a *showed no differentiation according to geographical location.

**Table 3:** Permutation tests for Hudson's statistic.

Gene	Subdivisions			Kst	Ks	Kt	P value^a^
*Avr3a*	A	vs	B/C	-0.023100	0.040122	0.039216	NS
	A	vs	V	nan	0.000	0.000	NS
	B/C	vs	V	-0.030565	0.043854	0.042553	NS
	N	*vs*	V	0.037646	1.844118	1.916256	NS
	N	*vs*	A	0.065266	1.589757	1.700758	NS
	N	*vs*	B/C	0.127264	0.999764	1.145551	NS

*Cox1*							
	A	vs	B/C	-0.021429	0.039286	0.038462	NS
	A	vs	V	0.856818	0.068182	0.476190	<0.001
	B/C	vs	V	0.774561	0.083859	0.371981	<0.001
	N	vs	V	0.487489	0.263577	0.514286	NS
	N	vs	A	0.131826	0.123671	0.142450	NS
	N	vs	B/C	0.093524	0.115370	0.127273	NS

*Ras*							
	A	vs	B/C	-nan	0.000	0.000	NS
	A	vs	V	0.300420	0.835913	1.194879	0.001
	B/C	vs	V	0.386997	0.557276	0.909091	<0.001
	N	vs	V	0.237184	1.015038	1.330645	NS
	N	vs	A	-nan	0.000	0.000	NS
	N	vs	B/C	-nan	0.000	0.000	NS

*β-tubulin*							
	A	vs	B/C	0.071097	0.443943	0.477922	0.001
	A	vs	V	0.022594	0.662711	0.678030	NS
	B/C	vs	V	0.002664	0.161134	0.161565	NS
	N	vs	V	0.082367	1.938315	2.112299	NS
	N	vs	A	0.075742	1.999553	2.163415	NS
	N	vs	B/C	0.178038	1.170111	1.423559	<0.001

*Phytophthora infestans *population subdivision was tested in the Northern Andean region for *Avr3a*, Cox1, *Ras *and *β-tubulin*.; V: Venezuela; A: Antioquia; N: Nariño; B: Boyacá; C: Cundinamarca.

**Table 4:** Neutrality test using Tajima's D

	Locus	Tajima's-D
**Northern Andean Region**	Cox1	0.54 (NS^a^)
	Ras	0.1 (NS)
	*β-tub*	-1.64(NS)
	Avr3a	-1.89 (<0.05)

*Cox1*, *Ras*, *β-tubulin *and *Avr3a *from *Phytophthora infestans *in the Northern

Andean region were tested for neutral evolution employing Tajima's D [42]. ^a^NS: Not significant

Tajima's D test failed to reject the null hypothesis of neutral evolution in *Ras*, *β*-*tubulin *and *Cox*1 (Table 4). This indicates that, for these loci, the Northern Andean population has not been subject to selection pressure. However, the *Avr3a *gene was found to be subject to a selection pressure. A negative tendency was observed in *Avr3a *and *β-tubulin *indicating a possible population expansion or a recent bottleneck effect. The R2 test failed to reject the null hypothesis of constant population size in the NA populations. The L shape pattern found in the mismatch distribution graphs of all genes (Additional file 6) is also indicative of a constant population size. No fixed differences were observed in any of the defined populations in the NA dataset (data not shown).

The analyses of gene flow were in general concordant between nuclear and mitochondrial genes (Table 5). Nuclear and mitochondrial genes showed low levels of genetic flow between Colombia and Venezuela. Both nuclear and mitochondrial genes showed that the Central and Eastern Andes are interchanging migrants.

**Table 5:** Migration and theta estimates for the Northern Andean populations of *Phytophthora infestans*.

	Theta	Number of Migrants per generation			
		Source population			
Recipient population		**Central ** **Andes**	**Eastern ** **Andes**	**Southwestern ** **Andes**	**Venezuela**
					
**Central Andes**	8.2e-3 (4.3e-3 - 0.019)	-	364.08 (147.23-1071.6)	8.4e-12 (3.85e-12 - 25.9)	8.4e-12 (3.85e-12 - 25.9)
	2e-4 (1e-4 - 3e-4)	-	4.08 (1.22 - 9.54)	6.8e-7 (3e-7 - 1.28e-3)	6.8e-7 (3e-7 - 1.28e-3)
					
**Eastern Andes**	8.8e-3 (7.6e-3 - 0.0102)	1.43e-14 (1.08e-14 - 0.81)	- -	1.43e-14 (1.08e-14-0.81)	1.43e-14 (1.08e-14-0.81)
	2e-4 (2e-4 - 2e-4)	2e-7 (3.2e-7 - 2.4e-4)		1.72e-1 (4.08e-2 - 4.6e-1)	1.68e+1 (1.49e+1 - 1.9 e+1)
					
**Southwestern Andes**	0.0048 (0.0023-0.0125)	19.97 (4.15 - 100.5)	43.97 (12.33-181.5)		5.14e-10 (2.16e-10 - 14.11)
	0.001 (0.0007-0.0018)	2.14 (1.16-7.92)	1e-6 (7e-7 - 2.38e-3)	-	2.62 (6.2e-1 - 9.22)
					
**Venezuela**	0,0057 (0.0033-0.0108)	24.39 (8.02-74.73)	2.76e-15 (1.41e-15 - 6.27)	4.89 (0.67-24.6)	-
	0.0002 (0.0001-0.0003)	2e-7 (1.6e-7 - 3.72e-4)	20 (8.94 - 34.1)	1.07e-1 (5.65e-3 - 5.86e-1)	

Migrations estimates correspond to migrants per generation (Nm) and 5% and 95%, confidence intervals are reported as are implemented in MIGRATE [45]. For each population the estimates correspond to the nuclear genes (above) and the mitochondrial gene (below).

### Avr3a Amino Acid Analysis

**Figure 3:**
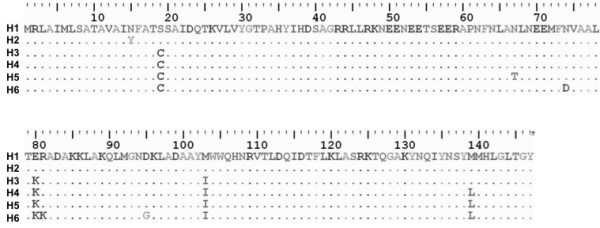
**Alignment of the Amino acid haplotypes observed at the Avr3a locus**. The six AVR3a haplotypes are shown with the shared amino acid positions represented by dots and replacements in IUPAC code.

Six different amino acid sequences were obtained after the translation of the nucleotide sequences from the W dataset (Figure 3). H1 was characterized by amino acids E80 and M103 and is associated with a virulent phenotype. H3, characterized by amino acids K80 and I103 and associated with an avirulent phenotype, was exclusively found in Ecuador, as was H4. To identify which amino acid positions were under diversifying selection, three pairs of ML models of amino acid substitutions were tested. Each pair consisted of one model that allowed sites to be under diversifying selection with ω > 1 (M2, M3 and M8) and a second model that did not allow sites to be under selection (M0, M1 and M7) with ω= dN/dS. The likelihood ratio tests failed to reject the models of neutral evolution. However, model M8 accounted for a small percentage (2%) of amino acids under selection pressure (ω >1). These amino acid positions were S19C, E80K and M103I.

## Discussion

This is the first time that a detailed population genetic analysis of *P. infestans *in the northern Andean region was conducted. Until now, the pathogen population structure was unknown in this portion of the continent (Colombia and Venezuela). Colombia is situated in a critical geographical position, acting as a bridge between Central and South America. According to FAO statistics, this country has a commercial history in trading potatoes with several countries in Europe, Asia, North and South America, and the Caribbean [24]. This suggests there have been opportunities for migration of the pathogen, along with its host, towards and within the northern Andean region.

The low genetic diversity found on global and regional scales in the Northern Andean region (Colombia and Venezuela), in both mitochondrial and nuclear regions, is consistent with previous reports in other countries of South America [8]. Even for microsatellite markers low levels of genetic diversity have been reported in Colombia and Venezuela [25-27]. Specifically, for *Ras*, only 2 out of 13 haplotypes found globally could be observed in the region. The A2 mating type was discovered in Ecuador and Colombia [16,26], and sexual reproduction is expected to produce new allele combinations. However, even though the A1 and A2 mating types converge in the same geographical region, as occurred in Cundinamarca Department (Colombia), sexual recombination is apparently not prevalent. This is probably due to a process of host adaptation [11,16,48], or because the presence of the A2 mating type is too recent to have resulted in recombination. Additionally, the low frequency of the A2 mating type described in Colombia diminishes the chances for sexual reproduction to occur [26]. Indeed, in the nuclear genes few individuals were heterozygous, suggesting that genetic interchange occurs but at extremely low frequency. Because of this, the presence of heterozygous individuals might be the result of ancestral polymorphisms or gene flow and not the result of sexual reproduction.

The gene networks for the different regions showed, as a general pattern, that the relationships between the haplotypes in each population of the Northern Andean region are consistent with variation within a clonal population and that haplotypes are discriminated by one or two steps. However, in the Southern Andes, at least for *β-tubulin *and *Avr3a*, more than two steps separated four and two haplotypes, respectively. The more divergent haplotypes were found in samples from *S. betaceum *and corresponded to the mitochondrial haplotype Ia. Recently it has been suggested that a new species, *P. andina*, with an Ia mitochondrial haplotype, could be associated with *S. betaceum *[8,23,49]. However, its taxonomic status has not been clarified yet, and continues to be considered as *P. infestans sensu lato*. The presence of this new species in the population of the southwestern Andes could explain the different pattern observed in *Avr3a *and *β-tubulin*.

The genetic variation of *P. infestans *is mainly explained by the variability present within each population that is generated by the existence of different low-frequency alleles, particularly for *β-tubulin *and Avr3a in the Southern Andes. Nevertheless, the variation detected in this study was enough to show some genetic differentiation. At the regional level, the *P. infestans *population in Venezuela appeared to be isolated from the Central and Eastern Andes populations. Indeed, at the mitochondrial level Venezuelan isolates belonged largely (11 out of 12) to the Ia haplotype, and just one corresponded to IIa, the most common haplotype in the North Andean region. This and the presence of a particular haplotypic composition for the nuclear genes may suggest that the Venezuelan population probably has been structured by different population events. Reasons for the apparent genetic isolation of the Venezuelan isolates of *P. infestans *have been discussed elsewhere [27]. The estimates of genetic flow support the idea that the population of Venezuela is not donating or receiving migrants from any other region (Table 5). However, a large area has not been sampled on the Colombian side close to the Venezuelan border. More sampling is therefore needed in this region in order to detect possible recent gene flow.

Additionally, two significant patterns were observed. First, subdivision was found between the Eastern Andes with the rest of Colombia for *β-tubulin*. This could be the result of a long history of self-sufficiency in terms of potato seed tuber supply in the eastern Andes as well as avoiding movement of the pathogen on plant tissue. Second, results obtained with the mitochondrial gene *Cox1 *and nuclear genes suggested that historically the Southwestern and the Eastern *P. infestans *populations have been isolated, but recent gene flow could be taking place.

The selection imposed on a gene with a known function in host recognition as *Avr3a *may have resulted in a different pattern of sequence polymorphisms in the sampled population in comparison to the other nuclear regions analyzed. However, low genetic diversity was found for this gene. No genetic subdivision could be detected in the Northern Andean region, in contrast with what was found for *β-tubulin *and *Ras*. At the amino acid level interesting patterns emerged. Previous studies reported only three polymorphic positions in a sampling of isolates from *S. tuberosum *from different locations worldwide. Two alleles were characterized based on those amino acid positions, C19 K80 I103 and S19 E80 M103, with avirulent and virulent phenotypes, respectively [28]. Here we report four new allelic variants. Three were only present in the isolates from the southwestern Andes. The natural selection analysis at the amino acid level showed that three out of the nine polymorphic positions were under diversifying selection and two of these were located in the C-terminus region as previously reported [28]. According to the model of interaction of the virulence genes with the host cell, at least two mechanisms could be affected by amino acid substitutions. The first one is the direct or indirect recognition by the resistance protein from the host. Bos et al. [50] have shown that some mutations leading to specific amino acid substitutions at the position E80K of this protein can be associated with a loss of recognition by the R3a gene product. On the other hand, the replacement of lysine by arginine at the same position of the protein does not affect the cell-death suppression activity of AVR3a [50]. It has been recognized that the variant AVR3aEM produces significantly less hypersensitive response than the other known variants of the gene, producing a virulent phenotype [28], while the AVR3aKI variant is the most effective in suppressing cell-death in the host [51]. This scenario leads to the possible advantage of maintaining polymorphic residues, which may give the pathogen population different adaptive pathways. The remaining polymorphic positions detected in this study were not under strong positive selection. However, their effect on avirulence on *S. tuberosum *R3-expressing plants remains to be determined in order to define their potential effect on host resistance at the subspecific level.

Polymorphisms of the *Avr3a *gene may be related to the plant species from which the isolates were collected. Armstrong et al. [28] reported that 55 isolates of *S. tuberosum *from different locations in the world contained only two alleles. In this study we found both previously reported haplotypes AVR3aEM (H1) in *S. tuberosum *as well as in other *Solanum *species, and AVR3aKI (H3) restricted to *S. muricatum*. The other four reported variants were not present in *S. tuberosum*. Because of this, it is required to establish the correlation of each of these variants and their host range. Additionally, the taxonomic status of the *P. infestans-*related species *P. andina *should be clarified in order to understand the real sympatry of the two species and their respective contribution of alleles to the population. Furthermore, a more intensive sampling is needed in order to correlate polymorphisms with the host. Finally, the wider picture of the population structure in this region suggests that the low steps in the networks, together with the low genetic diversity of this pathogen and the evidence of the presence of selection in the effector gene, might be consistent with a scenario of random dispersion of new mutations by drift that in some cases might be fixed by selection. We need further research in other populations and more data from effector genes and neutral loci to really understand the *Phytophthora infestans *diversification process in South America.

## Conclusions

The Northern Andean *P. infestans *population genetic analysis suggests that a clonal polymorphic population inhabits the region with population subdivision between Colombia and Venezuela as a result of potato seed self-sufficiency. Given the observed variation at an avirulence locus, the study of this type of loci could be useful to understand the epidemiological behavior of this pathogen on different hosts and in response to other ecological variables. Avirulence genes are thought to coevolve in an arms race with corresponding resistance genes in the host plant. Thus avirulence genes or other effectors could be excellent candidates for understanding the evolutionary forces responsible for the diversity observed, particularly for clonally reproducing populations of this pathogen. As was said before, comparisons of other populations and neutral vs. avirulence loci are needed to understand the population dynamics of *Phytopthora infestans*.

## Authors' contributions

MC acquired data, carried out and analyzed the molecular genetic studies, and drafted the manuscript. AG carried out the molecular genetic studies and drafted the manuscript. RS participated in acquisition of data and drafted the manuscript. CS participated in the molecular genetic analyses, and drafted and critically revised the manuscript. AR, AG, AV, MM, GF and LEL participated in acquisition of data and in revising the manuscript. NJG, AB, CS, and SR conceived the study, participated in its design and coordination, and helped to draft the manuscript. All authors read and approved the final manuscript.

## Supplementary Material

Additional file 1 ***Phytophthora infestans *isolates analyzed in this study**. Click here for file

Additional file 2 **Geographical distribution of the sampling sites**. Click here for file

Additional file 3 **Sequences retrieved from the GenBank public database employed in this study for *Ras*, *Cox1*, *β-tubulin *and ITS**. Click here for file

Additional file 4 **Gene networks showing relationships among haplotypes for each gene in each North Andean region**. Click here for file

Additional file 5 **Gene networks showing relationships among haplotypes for Colombia and Venezuela**. Click here for file

Additional file 6 **Mismatch distribution for all analyzed regions**. Click here for file
