# Supplementary material for: Genetic diversity of Phytophthora infestans in the Northern Andean region
Source: BMC Genet. 2011 Feb 9;12:23. doi: 10.1186/1471-2156-12-23 (PMC3046917; doi:10.1186/1471-2156-12-23)
Supplement: Additional file 1 — Phytophthora infestans isolates analyzed in this study. [file 1471-2156-12-23-S1.DOCX]

|  |  | **Country** | **Region** | **County** | **Year** | **International** | **Local** | | | |  | **mt** | **RAS** | **COX1** | **β-tub** | **Avr3a** |
| --- | --- | --- | --- | --- | --- | --- | --- | --- | --- | --- | --- | --- | --- | --- | --- | --- |
| 1 | *P. infestans* | Colombia | Antioquia | Rionegro | 2006 | 5011 (Vargas et al., 2009) | CO | A | SQ | 21 | *S. quitoenese* | IIa | 2 | 1 | 1 | 1 |
| 2 | *P. infestans* | Colombia | Antioquia | Rionegro | 2006 | 5021 (Vargas et al., 2009) | CO | A | SQ | 22 | *S. quitoenese* | IIa | 2 | 1 | 1 | 1 |
| 3 | *P. infestans* | Colombia | Antioquia | Rionegro | 2006 | 5031 (Vargas et al., 2009) | CO | A | SQ | 23 | *S. quitoenese* | IIa | 2 | 1 | 1/9 | 1 |
| 4 | *P. infestans* | Colombia | Antioquia | Rionegro | 2006 | 5041 (Vargas et al., 2009) | CO | A | SQ | 24 | *S. quitoenese* | IIa | 2 | 1 | 1 | 1 |
| 5 | *P. infestans* | Colombia | Antioquia | Rionegro | 2006 | 6011 (Vargas et al., 2009) | CO | A | SB | 25 | *S. betaceum* | IIa | 2 | 1 | 7/9 | 1 |
| 6 | *P. infestans* | Colombia | Antioquia | Rionegro | 2006 | 6021 (Vargas et al., 2009) | CO | A | SB | 27 | *S. betaceum* | IIa | 2 | 1 | 1 | 1 |
| 7 | *P. infestans* | Colombia | Antioquia | Rionegro | 2006 | 6041 (Vargas et al., 2009) | CO | A | SB | 30 | *S. betaceum* | IIa | 2 | 1 | 1 | 1 |
| 8 | *P. infestans* | Colombia | Antioquia | Rionegro | 2006 |  | CO | A | ST | 51 | *S. tuberosum* | *IIa* | 2 | 1 | 10 | N/D |
| 9 | *P. infestans* | Colombia | Antioquia | Rionegro | 2006 |  | CO | A | ST | 52 | *S. tuberosum* | *IIa* | 2 | 1 | 9 | 1 |
| 10 | *P. infestans* | Colombia | Antioquia | Rionegro | 2006 |  | CO | A | ST | 53 | *S. tuberosum* | *IIa* | 2 | N/D | 1 | 1 |
| 11 | *P. infestans* | Colombia | Antioquia | Rionegro | 2006 |  | CO | A | ST | 54 | *S. tuberosum* | *IIa* | 2 | 1 | 1 | 1 |
| 12 | *P. infestans* | Colombia | Antioquia | Rionegro | 2006 |  | CO | A | ST | 55 | *S. tuberosum* | *IIa* | 2 | 1 | 1 | 1 |
| 13 | *P. infestans* | Colombia | Antioquia | Rionegro | 2006 |  | CO | A | ST | 56 | *S. tuberosum* | *IIa* | 2 | 1 | 1 | 1 |
| 14 | *P. infestans* | Colombia | Antioquia | Rionegro | 2006 |  | CO | A | ST | 57 | *S. tuberosum* | *IIa* | 2 | 1 | 1 | 1 |
| 15 | *P. infestans* | Colombia | Antioquia | Rionegro | 2006 |  | CO | A | ST | 58 | *S. tuberosum* | *IIa* | 2 | 1 | 1 | 1 |
| 16 | *P. infestans* | Colombia | Antioquia | Rionegro | 2006 |  | CO | A | ST | 59 | *S. tuberosum* | *IIa* | 2 | 1 | 1 | 1 |
| 17 | *P. infestans* | Colombia | Antioquia | Rionegro | 2006 |  | CO | A | ST | 60 | *S. tuberosum* | *IIa* | 2 | 1 | 10 | N/D |
| 18 | *P. infestans* | Colombia | Antioquia | Rionegro | 2006 |  | CO | A | ST | 61 | *S. tuberosum* | *IIa* | 2 | 1 | 10 | 1 |
| 19 | *P. infestans* | Colombia | Boyacá | Toca | 2006 |  | CO | B | ST | 62 | *S. tuberosum* | *IIa* | 2 | 1 | 1 | 1 |
| 20 | *P. infestans* | Colombia | Boyacá | Toca | 2006 |  | CO | B | ST | 63 | *S. tuberosum* | *IIa* | 2 | 1 | 11 | 1 |
| 21 | *P. infestans* | Colombia | Boyacá | Toca | 2006 |  | CO | B | ST | 64 | *S. tuberosum* | *IIa* | 2 | 1 | 1 | 1 |
| 22 | *P. infestans* | Colombia | Boyacá | Toca | 2006 |  | CO | B | ST | 65 | *S. tuberosum* | *IIa* | 2 | 1 | 1 | 1 |
| 23 | *P. infestans* | Colombia | Boyacá | Toca | 2006 |  | CO | B | ST | 66 | *S. tuberosum* | *IIa* | 2 | 1 | 1 | 1 |
| 24 | *P. infestans* | Colombia | Boyacá | Toca | 2006 |  | CO | B | ST | 67 | *S. tuberosum* | *IIa* | 2 | 1 | 1 | 1 |
| 25 | *P. infestans* | Colombia | Boyacá | Toca | 2006 |  | CO | B | ST | 68 | *S. tuberosum* | *IIa* | 2 | 1 | 11 | 1 |
| 26 | *P. infestans* | Colombia | Boyacá | Toca | 2006 |  | CO | B | ST | 69 | *S. tuberosum* | *IIa* | 2 | 1 | 1 | 1 |
| 27 | *P. infestans* | Colombia | Boyacá | Toca | 2006 |  | CO | B | ST | 70 | *S. tuberosum* | *IIa* | 2 | 1 | 1 | 1 |
| 28 | *P. infestans* | Colombia | Boyacá | Toca | 2006 |  | CO | B | ST | 71 | *S. tuberosum* | *IIa* | 2 | 1 | 1 | 1 |
| 29 | *P. infestans* | Colombia | Boyacá | Toca | 2006 |  | CO | B | ST | 72 | *S. tuberosum* | *IIa* | 2 | 1 | 1 | 1 |
| 30 | *P. infestans* | Colombia | Boyacá | Toca | 2006 |  | CO | B | ST | 73 | *S. tuberosum* | *IIa* | 2 | 1 | 1 | 1 |
| 31 | *P. infestans* | Colombia | Cundinamarca | Zipaquirá | 2006 | 1061(Vargas et al., 2009) | CO | C | ST | 1 | *S. tuberosum* | IIa | 2 | 1 | 1 | 1 |
| 32 | *P. infestans* | Colombia | Cundinamarca | Cogua | 2006 | 1014 (Vargas et al., 2009) | CO | C | ST | 2 | *S. tuberosum* | IIa | 2 | 1 | 1 | 1 |
| 33 | *P. infestans* | Colombia | Cundinamarca | Zipaquirá | 2006 | 1016 (Vargas et al., 2009) | CO | C | ST | 3 | *S. tuberosum* | IIa | 2 | 1 | 1 | 1 |
| 34 | *P. infestans* | Colombia | Cundinamarca | Zipaquirá | 2006 | 1032 (Vargas et al., 2009) | CO | C | ST | 4 | *S. tuberosum* | IIa | 2 | 1 | 1 | 1 |
| 35 | *P. infestans* | Colombia | Cundinamarca | Cogua | 2006 | 1033 (Vargas et al., 2009) | CO | C | ST | 5 | *S. tuberosum* | IIa | 2 | 1 | 1 | 1 |
| 36 | *P. infestans* | Colombia | Cundinamarca | Cogua | 2006 | 1045 (Vargas et al., 2009) | CO | C | ST | 6 | *S. tuberosum* | IIa | 2 | 1 | 1 | 1 |
| 37 | *P. infestans* | Colombia | Cundinamarca | Cogua | 2006 | 2011 (Vargas et al., 2009) | CO | C | SP | 7 | *S. phureja* | IIa | 2 | 1 | 1 | 1 |
| 38 | *P. infestans* | Colombia | Cundinamarca | Cogua | 2006 | 2021 (Vargas et al., 2009) | CO | C | SP | 8 | *S. phureja* | IIa | 2 | 1 | 1 | 1 |
| 39 | *P. infestans* | Colombia | Cundinamarca | Cogua | 2006 | 2031 (Vargas et al., 2009) | CO | C | SP | 9 | *S. phureja* | IIa | 2 | 1 | 1 | 1 |
| 40 | *P. infestans* | Colombia | Cundinamarca | Cogua | 2006 | 2061 (Vargas et al., 2009) | CO | C | SP | 10 | *S. phureja* | IIa | 2 | 1 | 1 | 1 |
| 41 | *P. infestans* | Colombia | Cundinamarca | Villapinzón | 2006 | 3012 (Vargas et al., 2009) | CO | C | SL | 11 | *S. lycopersicum* | IIa | 2 | 1 | 1 | 1 |
| 42 | *P. infestans* | Colombia | Cundinamarca | Fusagasugá | 2006 | 3013 (Vargas et al., 2009) | CO | C | SL | 12 | *S. lycopersicum* | IIa | 2 | 1 | 1 | 1 |
| 43 | *P. infestans* | Colombia | Cundinamarca | Tibacuy | 2006 | 3025 (Vargas et al., 2009) | CO | C | SL | 13 | *S. lycopersicum* | IIa | 2 | 1 | 1 | 1 |
| 44 | *P. infestans* | Colombia | Cundinamarca | Bogotá | 2006 | 3034 (Vargas et al., 2009) | CO | C | SL | 14 | *S. lycopersicum* | IIa | 2 | 1 | 1 | 1 |
| 45 | *P. infestans* | Colombia | Cundinamarca | Tibacuy | 2006 | 3035 (Vargas et al., 2009) | CO | C | SL | 15 | *S. lycopersicum* | IIa | 2 | 1 | 1/9 | 1 |
| 46 | *P. infestans* | Colombia | Cundinamarca | San Francisco | 2006 | 4012 (Vargas et al., 2009) | CO | C | PP | 16 | *P. peruviana* | IIa | 2 | 1 | 1 | 1 |
| 47 | *P. infestans* | Colombia | Cundinamarca | San Francisco | 2006 | 4013 (Vargas et al., 2009) | CO | C | PP | 17 | *P. peruviana* | IIa | 2 | 1 | 1 | 1 |
| 48 | *P. infestans* | Colombia | Cundinamarca | Granada | 2006 | 4024 (Vargas et al., 2009) | CO | C | PP | 18 | *P. peruviana* | IIa | 2 | 1 | 1 | 1 |
| 49 | *P. infestans* | Colombia | Cundinamarca | Granada | 2006 | 4034 (Vargas et al., 2009) | CO | C | PP | 19 | *P. peruviana* | IIa | 2 | 1 | 1 | 1 |
| 50 | *P. infestans* | Colombia | Cundinamarca | Granada | 2006 | 4064 (Vargas et al., 2009) | CO | C | PP | 20 | *P. peruviana* | IIa | 2 | 1 | 1 | 1 |
| 51 | *P. infestans* | Colombia | Cundinamarca | Granada | 2006 | 6012 (Vargas et al., 2009) | CO | C | SB | 26 | *S. betaceum* | IIa | 2 | 1 | 1 | 1 |
| 52 | *P. infestans* | Colombia | Cundinamarca | Granada | 2006 | 4084 (Vargas et al., 2009) | CO | C | PP | 28 | *P. peruviana* | Ia | 2 | 5 | 1 | 1 |
| 53 | *P. infestans* | Colombia | Cundinamarca | Granada | 2006 | 6022 (Vargas et al., 2009) | CO | C | SB | 29 | *S. betaceum* | IIa | 2 | 1 | 1 | 2 |
| 54 | *P. infestans* | Colombia | Nariño | Genoy | 2005 |  | CO | N | SB | 31 | *S. betaceum* | Ia | 2 | 1 | 5 | 1 |
| 55 | *P. infestans* | Colombia | Nariño | Pasto | 1999 |  | CO | N | ST | 34 | *S. tuberosum* | IIa | 2 | 1 | 8/9 | 1 |
| 56 | *P. infestans* | Colombia | Nariño | Pasto | 1999 |  | CO | N | ST | 35 | *S. tuberosum* | IIa | 2 | N/D | N/D | 1 |
| 57 | *P. infestans* | Colombia | Nariño | Pasto | 1999 |  | CO | N | ST | 36 | *S. tuberosum* | IIa | 2 | N/D | 1 | 1 |
| 58 | *P. infestans* | Colombia | Nariño | Pasto | 1999 |  | CO | N | ST | 37 | *S. tuberosum* | IIa | 2 | N/D | 1 | 1 |
| 59 | *P. infestans* | Colombia | Nariño | Pasto | 1999 |  | CO | N | ST | 38 | *S. tuberosum* | IIa | 2 | 1 | 1/9 | 1 |
| 60 | *P. infestans* | Colombia | Nariño | Pasto | 1999 |  | CO | N | SP | 39 | *S. phureja* | IIa | 2 | 1 | 1/9 | 1 |
| 61 | *P. infestans* | Colombia | Nariño | Pasto | 1999 |  | CO | N | ST | 40 | *S. tuberosum* | IIa | N/D | 1 | 1/9 | 1 |
| 62 | *P. infestans* | Colombia | Nariño | Pasto | 1999 |  | CO | N | ST | 41 | *S. tuberosum* | IIa | N/D | N/D | 1/7 | 1 |
| 63 | *P. infestans* | Colombia | Nariño | Pasto | 1999 |  | CO | N | ST | 43 | *S. tuberosum* | IIa | 2 | N/D | 8/9 | 1 |
| 64 | *P. infestans* | Colombia | Nariño | Pasto | 1999 |  | CO | N | ST | 44 | *S. tuberosum* | IIa | 2 | 1 | 1/7 | 1 |
| 65 | *P. infestans* | Colombia | Nariño | Genoy | 2003 |  | CO | N | SB | 45 | *S. betaceum* | Ia | 2 | 5 | 3 /4 | 1 |
| 66 | *P. infestans* | Colombia | Nariño | Buesaco | 2003 |  | CO | N | SB | 48 | *S. betaceum* | Ia | 2 | 1 | 2/6 | 1 |
| 67 | *P. infestans* | Colombia | Nariño | San Bernardo | 2004 |  | CO | N | SB | 49 | *S. betaceum* | Ia | N/D | 1 | N/D | 5/6 |
| 68 | *P. infestans* | Colombia | Nariño | Obonuco | 2003 |  | CO | N | SB | 50 | *S. betaceum* | Ia | 2 | 5 | N/D | 5/6 |
| 69 | *P. infestans* | Venezuela | Mérida | Santa Rosa | 2004 | V1  Briceño et al., 2009 |  | V | ST | 86 | *S. tuberosum* | Ia | 2/13 | 5 | 1 | 1 |
| 70 | *P. infestans* | Venezuela | Táchira | Las Porqueras | 2005 | V2  Briceño et al., 2009 |  | V | ST | 87 | *S. tuberosum* | IIa | 2/13 | 1 | 1 | 1 |
| 71 | *P. infestans* | Venezuela | Mérida | El Valle | 2004 | V3  Briceño et al., 2009 |  | V | ST | 88 | *S. tuberosum* | Ia | N/D | 5 | 1 | 1 |
| 72 | *P. infestans* | Venezuela | Mérida | Pueblo Llano | 2004 | V4  Briceño et al., 2009 |  | V | ST | 89 | *S. tuberosum* | Ia | 2/13 | 5 | 1/8 | 1 |
| 73 | *P. infestans* | Venezuela | Mérida | Pueblo Llano | 2004 | V5  Briceño et al., 2009 |  | V | ST | 90 | *S. tuberosum* | Ia | 2/13 | 5 | 1 | 1 |
| 74 | *P. infestans* | Venezuela | Mérida | Pueblo Llano | 2005 | V6  Briceño et al., 2009 |  | V | ST | 91 | *S. tuberosum* | Ia | N/D | 5 | 1 | 1 |
| 75 | *P. infestans* | Venezuela | Táchira | El Cobre | 2005 | V7  Briceño et al., 2009 |  | V | ST | 92 | *S. tuberosum* | Ia | 2/13 | 5 | 1 | 1 |
| 76 | *P. infestans* | Venezuela | Mérida | Valle del Chama | 2005 | V8  Briceño et al., 2009 |  | V | ST | 93 | *S. tuberosum* | Ia | 2/13 | 5 | 1 | 1 |
| 77 | *P. infestans* | Venezuela | Mérida | Valle del Mocotíes | 2005 | V9  Briceño et al., 2009 |  | V | ST | 94 | *S. tuberosum* | Ia | 2/13 | 5 | 1 | 1 |
| 78 | *P. infestans* | Venezuela | Trujillo | Valle del Tuñame | 2005 | V10  Briceño et al., 2009 |  | V | ST | 95 | *S. tuberosum* | Ia | 2/13 | 5 | 1 | 1 |
| 79 | *P. infestans* | Venezuela | Mérida | El Trompillo | 2005 | V11  Briceño et al., 2009 |  | V | ST | 96 | *S. tuberosum* | Ia | 2/13 | N/D | 1 | 1 |
| 80 | *P. infestans* | Venezuela | Mérida | Santo Domingo | 2004 | V12  Briceño et al., 2009 |  | V | ST | 97 | *S. tuberosum* | Ia | 2/13 | 5 | 1 | 1 |
| 81 | *P. infestans* | Ecuador |  | Tungurahua | 2004 | P13809 |  | E | SQ | 75 | *S. quitoense* | *Ia* | 2 | 5 | 7 | 4 |
| 82 | *P. infestans* | Ecuador |  | Pinchincha | 2003 | P13626 |  | E | ST | 76 | *S. tuberosum* | *IIa* | 2 | 1 | N/D | 1 |
| 83 | *P. infestans* | Ecuador |  | Tungurahua | 2001 | P13381 |  | E | SL | 78 | *S. lycopersicum* | *IIa* | 2 | 1 | N/D | 1 |
| 84 | *P. infestans* | Ecuador |  | Pinchincha | 1997 | P13150 |  | E | SM | 79 | *S. muricatum* | *Ia* | 2 | 5 | 7 | 3 |
| 85 | *P. infestans* | Ecuador |  | Napo | 2001 | P13444 |  | E | SC | 80 | *S. colombianum* | *IIa* | 2 | 1 | N/D | 1 |
| 86 | *P. infestans* | Ecuador |  | Tungurahua | 2001 | P13352 |  | E | ST | 81 | *S. tuberosum* | *IIa* | 2 | 1 | 1 | 1 |
| 87 | *P. infestans* | Ecuador |  | Carchi | 2005 | P13852 |  | E | SO | 84 | *S. ochrantum* | *IIa* | 2 | 1 | 1 | 1 |
| 88 | *P. infestans* | Ecuador |  | Azuay | 2006 | P13665 |  | E | SM | 85 | *S. muricatum* | *Ia* | 2 | 5 | 7 | 3 |
|  | | | | | | | | | | | | | | | | |
