# Supplementary figures and images for: Genetic diversity of Phytophthora infestans in the Northern Andean region
Source: BMC Genet. 2011 Feb 9;12:23. doi: 10.1186/1471-2156-12-23 (PMC3046917; doi:10.1186/1471-2156-12-23)

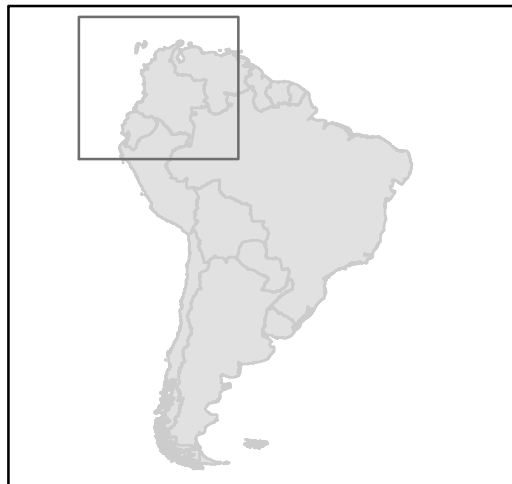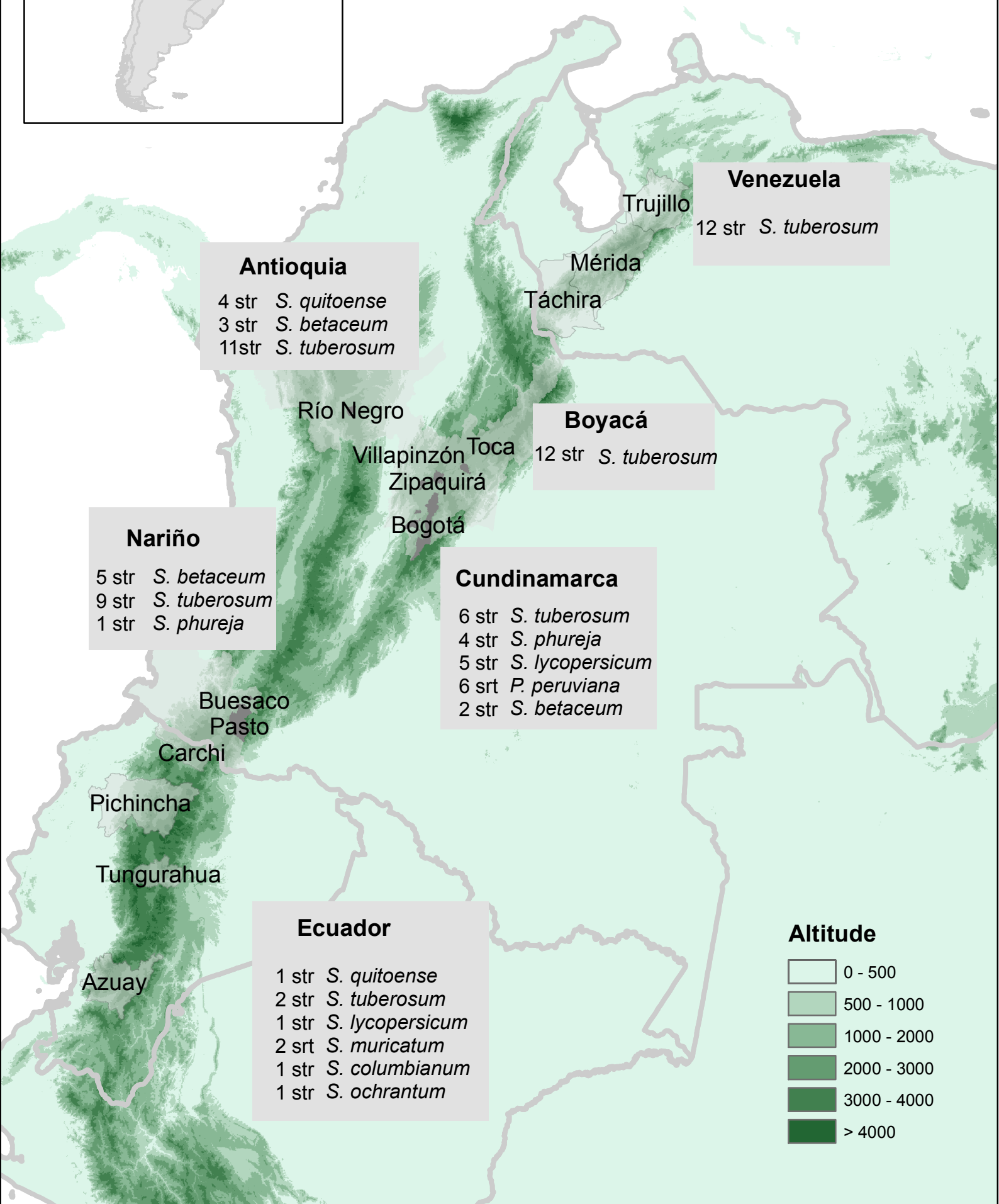

Supplement: Additional file 2 — Geographical distribution of the sampling sites. [file 1471-2156-12-23-S2.PDF]

Avr3a

B-TUB

Ras

Cox1

BC

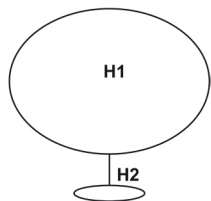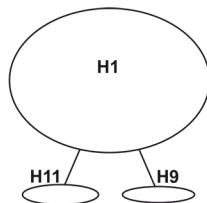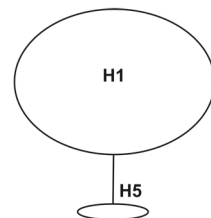

A

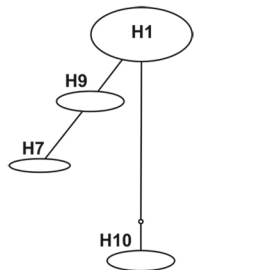

N

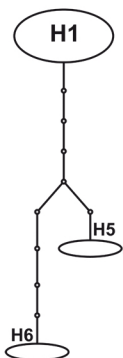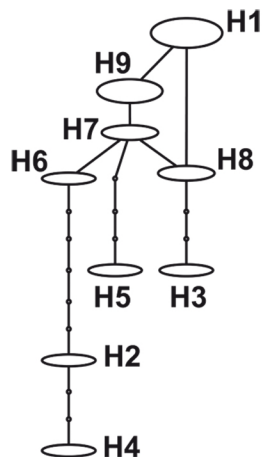

V

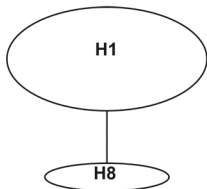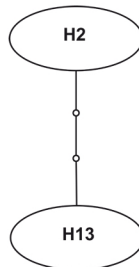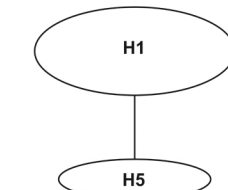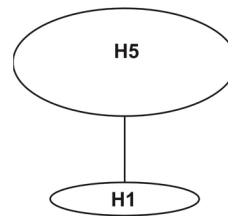

Supplement: Additional file 4 — Gene networks showing relationships among haplotypes for each gene in each North Andean region. [file 1471-2156-12-23-S4.PDF]

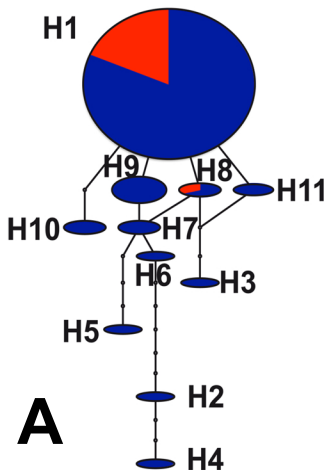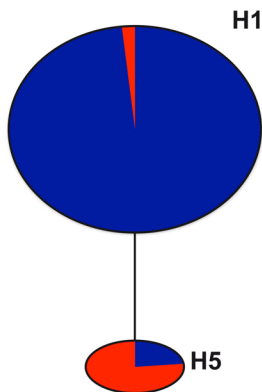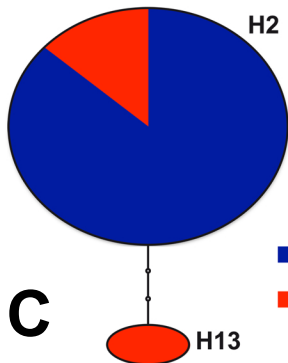

■ COLOMBIA  
■ VENEZUELA

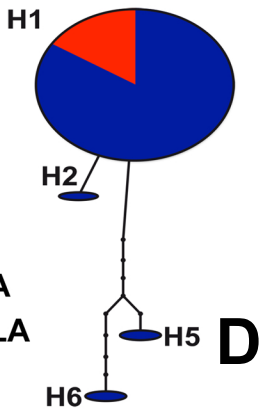

Supplement: Additional file 5 — Gene networks showing relationships among haplotypes for Colombia and Venezuela. [file 1471-2156-12-23-S5.PDF]

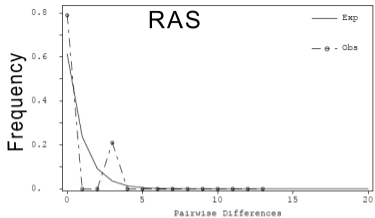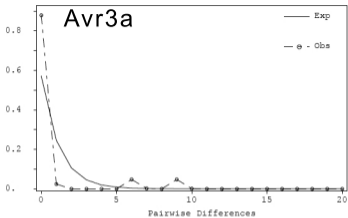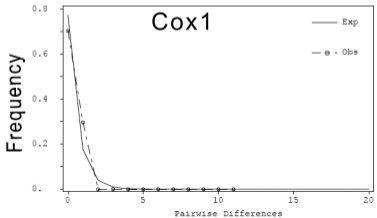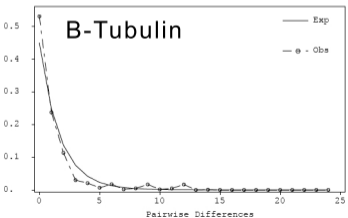

Supplement: Additional file 6 — Mismatch distribution for all analyzed regions. [file 1471-2156-12-23-S6.PDF]
