# Supplementary material for: Genetic diversity of Phytophthora infestans in the Northern Andean region
Source: BMC Genet. 2011 Feb 9;12:23. doi: 10.1186/1471-2156-12-23 (PMC3046917; doi:10.1186/1471-2156-12-23)
Supplement: Additional file 3 — Sequences retrieved from the GenBank public database employed in this study for Ras, Cox1, β-tubulin and ITS. [file 1471-2156-12-23-S3.DOCX]

### Additional file 2. Sequences retrieved from the GenBank public database employed in this study for *Ras-IRas*, *Cox1, β-tubulin* and ITS.

| GENETIC REGION | ACCESSION NUMBERS |
| --- | --- |
| *Ras* | EF367106.1- EF366951.1 |
| *IRas* | EF366950.1- EF366795.1 |
| *Cox1* | EF366794.1- EF366747.1  EF366744.1- EF366733.1 |
| *β-tubulin* | EU079633.1, EU079626.1, EU079613.1, AY564037.1, AY564036.1, AY564035.1 |
| ITS | DQ479409.1, EF126351.1, AY922974.1, AY770739.1, AY770731.1, AF489701.1, AF228083.1, AY829468.1, AY829464.1, AF339424.1, AF339422.1 |
